# Stigma, confidence, attitudes, barriers and incentive factors in pharmaceutical care of patiens with depression in Lithuania: a protocol for a prospective 3 years follow-up study

**DOI:** 10.1192/j.eurpsy.2023.1777

**Published:** 2023-07-19

**Authors:** M. Žūkaitė, J. Peceliuniene

**Affiliations:** 1 Pharmacy center; 2 clinic of internal diseases, family medicine and oncology; Pharmacy center, Vilnius University, Faculty of Medicine, Vilnius, Lithuania

## Abstract

**Introduction:**

According to the WHO, approximately 280 million people in the world have depression. It is known that pharmacists are in an ideal position to offer proactive interventions to people with depression or depressive symptoms. However, pharmacists’ stigmatizing attitudes towards depression and patients with mental illness may decrease the quality of pharmaceutical care services provided to those patients. No research has been conducted on pharmaceutical care and pharmacists attitudes towards patients with depression in Lithuania.

**Objectives:**

The aim of the study is to evaluate stigma, confidence, attitudes, barriers and incentive factors in providing pharmaceutical care to patients with depression in Lithuania.

**Methods:**

The prospective 3 years follow-up study will be carried out among the pharmacists in Lithuania. The sample size of 269 respondents is calculated. First of all, pharmacists will be provided with a training lecture by trained investigator (M.Z.) about depression and its pharmaceutical care. At the same day, after the lecture, pharmacists‘ stigma, confidendence, attitudes, barries and incentive factors in providing pharmaceutical care to patients with depression will be evaluated by 5 questionnaires:

1. Participants will be asked to provide sociodemographic information included age, gender, years of practice, etc;
2. The pharmacists’ stigma of patients with depression will be evaluated using eight likert-scale items that measure how patients with depression are perceived;
3. Pharmacists’ attitudes toward depression will be evaluated using Depression Attitude Questionnaire;
4. Pharmacists’ confidence in medication consultation for patients with depression will be evaluated using The Pharmacists’ Confidence scale about Medication Consultation for Depressive patients (PCMCD);
5. A list of possible barries and incentive factors identified in the literature will be provided to pharmacists and they will be asked to choose as many barriers and incentive factors as they think are relevant.

Next trainings lectures will be performed repeatedly at the month 6, 12, 18, 24, 30 and at the end of the study - month 36. Also, pharmacists‘ position will be re-evaluated after each training lecture by the same 5 questionnaires.

The Lithuanian Bioethics Commitee approval is going to be received after the training program is confirmed (estimated time – the 1st quarter of 2023).

**Results:**

Stigma, confidence, attitudes, barriers and incentive factors in providing pharmaceutical care to patients with depression in Lithuania will be evaluated.

**Conclusions:**

Conclusions will be drawn on stigma, confidence, attitudes, barriers and incentive factors in pharmaceutical care of patiens with depression in Lithuania. Also, practical recommendations will be introduced to The Ministry of Health of The Republic of Lithuania.

**Disclosure of Interest:**

None Declared

